# Stone in the Bladder

**Published:** 1880-07

**Authors:** R. F. Weir

**Affiliations:** Lecturer of Diseases of the Genito-Urinary Organs


CHICAGO MEDICAL

JOURNAL & EXAMINER.

Vol. XLI.—JULY, 1880.—No. 1.

### Original Lectures.

Article I.

Stone in the Bladder. A Clinical Lecture delivered at the College of Physicians and Surgeons of New York, by Dr. R. F. Weir, Lecturer of Diseases of the Genito-Urinary Organs.

What I have to say to-day is on the subject of stone. I have already shown you the production of ammonia in alkaline urine and how the changed urine allows the earthy salts to be deposited, which, uniting with the mucus of the bladder, may result in the formation a calculus. This form of stone may be called the inflammatory stone. But there are calculi which may arise from a urine too heavily laden with salts, as is seen in the summer time or when we catch cold, when the water and the phosphates are often thrown down as a red, or pinkish, deposit. We are more likely, however, to have this taking place in the kidney than in the bladder. There are, besides the usual constituents of the urine, certain other substances, such as xanthin and cystine, which occasionally constitute the nucleus of a calculus, but of all the various salts and organic deposits, uric acid and its compounds are by far the most frequent nuclei of stone.

Some years ago, an analysis of the nuclei of some sixty-two cases of stone was made by Mr. Carter, at that time a surgeon of India. He found that out of the sixty-two calculi, thirty-four were formed of urates; twenty-one of oxalates; seven of uric acid, pure. This was in India. When he came to England he then made another analysis, but this proportion did not obtain there, the uric acid compounds constituting 71 per cent. of all the stones, and the oxalates going down in the scale to 29 per cent., as against 56 per cent. uric acid compounds to 44 per cent of oxalates, in India. It is evident that the difference is owing to a difference in diet, the articles of food in India being largely farinaceous and thus producing more oxalates of lime.

Why then, if we are subject to deposits of salts such as these in the bladder, do we not all have stone? The fact that these deposits go to the formation of stone is not the only reason. Another condition is necessary. Carter found some peculiar rhomboidal crystals of urates and oxalates which he had not seen before, which were explained by the researches of Messrs. Ord and Ranney. They found that such submorphous or molecular forms of crystallization resulted from the presence of a certain gluey or colloid material in the bladder. They could be formed by placing two solutions, one of oxalate of potash, one of chloride of calcium, in a test tube, separated by a solidified solution of gelatine, when in the gelatine solution the same peculiar form of crystallization were to be seen which he had found in the urine, in the presence of the colloid or gelatinous matter, so he came to the conclusion that the presence of this colloid material in the bladder was essential to the production of a stone.

This tells us why these conditions occur in one kidney and not in another and why it is that calculi occur so frequently in early life (the must common period, except in adults past the age of 55 the subjects of bladder inflammation). Children are more apt to have temporary congestion of the kidneys, and when this takes place, this gluey material is poured out and we sometimes find, if we examine the urine, hyalin tubular casts, consisting of their colloid matter—casts, however, which are not of much importance luckily. When this colloid material, entangling some salts, happens, usually a healthy stream of urine breaks it down and washes it away. However, sometimes the nucleus may stay and grows into a calculus of increased size. When such a nucleus does take up its residence, either in the kidney or in the bladder, the inflammation which it excites as a foreign body causes an increased secretion of mucus, which, mingling with the urine, induces fermentation. The urine then becomes alkaline, and the earthy phosphates are deposited around the nucleus, causing the whitish layer so often seen in calculi. Here, however is a calculus in the kidney which is quite unique. It is a cystine one, weighing 175 gr. Cystine calculi are rare. This constituent of urine differs from the others, in that it has sulphur in it, which the others have not. It is this which gives it its peculiar character. When cut open such a calculus is soft, greasy, yellowish in color and fractures easily.

When a stone is formed in the kidney it may remain there for years, or it may ulcerate into or out of the pelvis, of the kidney, or it may stay in the ureter or get into the bladder, growing very large sometimes in both localities. The largest on record, found in the bladder, weighed six pounds. Such large stones are grasped by the bladder, so that the sound cannot pass it. Fifteen ounces is the weight of the largest stone that has been removed by operation. Here is one in the kidney weighing 54 ounces. I have said that a stone may remain in the kidney for an indefinite length of time. When that is the case the patient complains of vague pains in the region of the loins. He will suffer from increased frequency of micturition. His urine may be tinged with blood, evenly disseminated through the entire quantity. These will be all the symptoms, which are confessedly obscure, unless the stone pass out of the kidney into the ureter. In that case there will be intense pain in the loins from distension of the ureter, extending around the belly and into the testicle. Vomiting is also sometimes present. These symptoms may last from a few hours to several days, and then suddenly pass away. If you are called to such a patient and have relieved him by hypodermic injections of morphine, with which you ought to combine 1-100 of a grain of atropine, you must carefully inspect the urine for several days to see if the renal calculus has been passed per urethram. If it should stop in the ureter and there stay and your patient have another attack, and that on the other side, suppression of urine might take place. Roberts, of Manchester, has seen a number of cases of this sort. In such cases the specific gravity of the urine goes down to 1.002, 1.003 or 1.004. When there is pressure on the kidney, but little urine filters through and there is a diminution of the urea. How can such a condition of things be appreciated other than by these symptoms? You may explore the ureter through the rectum, and if the stone could be felt at one of its usual stopping-places, just at the bladder, it might be squeezed through the ureter into the bladder. If that condition did not exist, I do not think I should hesitate to make an incision into the loin and if the calculus were found at its other favorite place of arrest, within four inches of the kidney, to extract it and run the risk of making a urinary fistula and so perhaps save life. Something is to be said regarding the preventive treatment of renal calculi. It may arise, as I have stated, from an increased quantity of salts in the urine. Sedentary habits or indigestion may increase the amount of salts in the urine. These conditions are to be corrected. In certain regions of the country more cases of stone seem to arise than in others—notably the limestone regions, but in this case I do not think that it is the amount of lime taken into the system (which is very small) that occasions stone, but the fact that the lime salts interfere with digestion and so set up, through impaired retrograde metamorphoses, this condition before shaken off, namely a urine too heavily laden with salts.

After cholera epidemics it has been noticed that there will come an epidemic of stone in the bladder. In that case there is a thickened condition of the blood, the loss of the watery constituents being the result of the diarrhœa. In that way the circulation is laden with concentrated material and gives rise to this same excess of salts in the urine. Such a condition is also found in people addicted to the use of malt liquors and in the gouty diathesis. You will make such patients take plenty of exercise. Let them keep their skin in good condition so that too much labor may not fall to the kidneys. Can we do anything else? I think we can. Tell such a patient to avoid concentration of urine. At night time it is most concentrated and it remains in the tubules longest. You know that when you wish a specimen of urine for examination you always order the urine which has been passed first in the morning to be brought to you. That is because it is most concentrated. Your patient then should take a tumbler of water on going to bed, so that the urine in the tubules may be washed into the bladder, even if he has to get up to urinate towards morning. For medication, you may order the citrate of potash to be given, in doses of from forty to sixty grains three times a day. This can be taken for a long time without deranging the digestion. The Richfield Springs water in this country and the Carlsbad in Europe is very useful in maintaining a neutral condition of the urine. They owe their alkalinity to the sulphate of soda and potassa which they contain. They render the urine alkaline by virtue of these salts and do away with the clogged action of the liver, which is often the starting point of a calculus. By means of alkalies and purgatives we may hope to rid the patient of his troubles. The citrate of potash should be used fresh, as when kept it deteriorates. Natural mineral waters are preferable to the artificial. Sir Henry Thompson of London says that the natural waters do their work better than the artificial, although he cannot explain why. In the absence of the natural water the salts which are obtained from the natural waters by evaporation may be used. A teaspoonful of Carlsbad salts may be added to a tumbler of water and drank before breakfast in the morning. If taken warm, its purgative action is quickened. There are other useful mineral waters which you may rely on for treatment in these disorders. There is the Friedrichshall, which contains 58 per cent. of the sulphate of soda, and the Marienbad. But there is a water which is much more effective than any of these—the Hunyadi Janos. It is more pleasant to take than the bitter water of Friedrichshall and contains in 10,000 parts, 225 grains of the sulphate of soda and 223 grains sulphate of magnesia. It is fully twice the strength of any of the others. If your patient cannot buy the waters he can use Glauber's salts, which act nearly in the same way. Let him dissolve a teaspoonful of the salts in a tumbler of water.

Let it stand over night and drink in the morning. Increase the quantity if this does not act, for otherwise gas will accumulate in the stomach and bowels and cause distension, colicky pains and unpleasant rumblings. I have spoken of the citrate of potash before and told you that, as kept in the shops, it is often inert. Perhaps the best way to administer it is to make it extemporaneously by the reaction of citric acid and the bicarbonate of potash This mixture may be taken while effervescing.

So, when you can, let the patient take the natural mineral waters, for they are the best, but if they are beyond his means, you will have to fall back on these preparations, and they will do you good service.

---

ANTISEPTICS IN OPHTHALMIC SURGERY.—Galezowski (*Recueil d'Ophthalmologie*, November, 1879) recommends antiseptic precautions for most operations on the eye, principally, however, in enucleation, operations on the lids, etc., and cataract extraction. All the instruments used, as well as the sutures and sponges, should be dipped in a solution of carbolic acid, 1 to 1000(!), and the wound, as well as the skin round about, washed with the same solution. More concentrated solutions may be used in the case of enucleation, and the compresses must also be carbolized, etc. Galezowski also uses other antiseptic applications, such as boracic acid and vaseline, 1 to 100, in abscess of the cornea, also a 1 per cent, solution of boracic acid for the purification of instruments to be used in cataract operations. Galezowski has had most excellent results since he has begun his antiseptics. It is difficult to see how failures could occur at all with such precautions.(!) This subject was also discussed at the Amsterdam Congress, where it was introduced by Snellen, who uses a one per cent. solution of carbolic acid. He finds a spray impracticable, and has used instead, with great success, a current of air purified by being caused to pass through carbolic acid. As a dressing he uses linen saturated with vaseline, as he finds that the usual antiseptic dressings are too irritating, and causes an increased secretion from the conjunctiva and palpebral glands.

